# Ventilatory responses during and following hypercapnic gas challenge are impaired in male but not female endothelial NOS knock-out mice

**DOI:** 10.1038/s41598-021-99922-5

**Published:** 2021-10-18

**Authors:** Paulina M. Getsy, Sripriya Sundararajan, Walter J. May, Graham C. von Schill, Dylan K. McLaughlin, Lisa A. Palmer, Stephen J. Lewis

**Affiliations:** 1 Department of Pediatrics, Case Western Reserve University, Biomedical Research Building BRB 319, 10900 Euclid Avenue Mail Stop 1714, Cleveland, OH 44106-1714 USA; 2 Department of Physiology and Biophysics, Case Western Reserve University, Cleveland, OH USA; 3 Pediatric Respiratory Medicine, University of Virginia School of Medicine, Charlottesville, VA USA; 4 Department of Pharmacology, Case Western Reserve University, Cleveland, OH USA; 5 Functional Electrical Stimulation Center, Case Western Reserve University, Cleveland, OH USA; 6 Present Address: Division of Neonatology, Department of Pediatrics, University of Maryland School of Medicine, Baltimore, MD 21201 USA

**Keywords:** Neuroscience, Physiology, Systems biology

## Abstract

The roles of endothelial nitric oxide synthase (eNOS) in the ventilatory responses during and after a hypercapnic gas challenge (HCC, 5% CO_2_, 21% O_2_, 74% N_2_) were assessed in freely-moving female and male wild-type (WT) C57BL6 mice and eNOS knock-out (eNOS-/-) mice of C57BL6 background using whole body plethysmography. HCC elicited an array of ventilatory responses that were similar in male and female WT mice, such as increases in breathing frequency (with falls in inspiratory and expiratory times), and increases in tidal volume, minute ventilation, peak inspiratory and expiratory flows, and inspiratory and expiratory drives. eNOS-/- male mice had smaller increases in minute ventilation, peak inspiratory flow and inspiratory drive, and smaller decreases in inspiratory time than WT males. Ventilatory responses in female eNOS-/- mice were similar to those in female WT mice. The ventilatory excitatory phase upon return to room-air was similar in both male and female WT mice. However, the post-HCC increases in frequency of breathing (with decreases in inspiratory times), and increases in tidal volume, minute ventilation, inspiratory drive (i.e., tidal volume/inspiratory time) and expiratory drive (i.e., tidal volume/expiratory time), and peak inspiratory and expiratory flows in male eNOS-/- mice were smaller than in male WT mice. In contrast, the post-HCC responses in female eNOS-/- mice were equal to those of the female WT mice. These findings provide the first evidence that the loss of eNOS affects the ventilatory responses during and after HCC in male C57BL6 mice, whereas female C57BL6 mice can compensate for the loss of eNOS, at least in respect to triggering ventilatory responses to HCC.

## Introduction

The ventilatory responses to changes in arterial pO_2_, pCO_2_ and H^+^ ions are mediated by peripheral structures, such as the carotid bodies, and central structures within the brainstem, such as the retrotrapezoid nucleus (RTN)^1–5^. Chemosensitive glomus cells within the carotid bodies (CB) depolarize in response to decreases in blood pO_2_, increases in blood pCO_2_, and from carbonic anhydrase-catalyzed production of H^+^ ions from increased availability of blood CO_2_^6–13^. Depolarization activates the glomus cells causing them to release an array of excitatory neurotransmitters (e.g., ATP and acetylcholine)^14–24^ that trigger action potentials in the terminals of chemoafferents fibers, which send projections to the nucleus tractus solitarius (NTS) via the carotid sinus nerve (CSN)^6,8–12^. CSN chemoafferent input to the NTS signals a series of downstream cardiorespiratory responses, such as increases in minute ventilation and blood pressure, in order to restore arterial blood gas homeostasis^11,19,25^.

The involvement of CB chemoafferents in mediating the ventilatory responses to hypercapnic (HCC) and hypoxic (HXC) gas challenges have been explored in many species, including humans^26–34^, mice^35–37^, rats^38–52^ and dogs^53–55^. Hypoxic ventilatory signaling is vitally dependent upon the carotid body-carotid sinus nerve complex, whereas hypercapnic signaling is dependent on the carotid body-carotid sinus nerve complex^1,3,54,57,58^ and brainstem structures, including the RTN^59–66^. Wild-type (WT) mice of various strains and genetically engineered mice are used to investigate mechanisms by which HCC and HXC elicit ventilatory responses that are both dependent and independent of the carotid bodies^20,21,37,67–81^. We reported that the ventilatory responses elicited by a HXC were markedly reduced in freely-moving adult male C57BL6 mice with bilateral carotid sinus nerve transections^37^. Earlier, Izumizaki et al^51^ examined the involvement of the carotid body-carotid sinus nerve complex in breathing responses to a hyperoxic (100% O_2_)-hypercapnic (5% CO_2_) gas challenge in urethane-anesthetized adult male C57BL-6CrSlc mice, and found that the ventilatory responses were markedly reduced after bilateral carotid sinus nerve transection. It should also be noted that the increases in breathing in response to HCC and HXC often far out-last the period of gas exposure and these patterns of responses are collectively referred to as short-term potentiation (STP) of ventilation^37,73,82–85^.

In this study we used C57BL6 mice because the ventilatory and cardiovascular processes in this strain have been studied extensively, and this strain is widely used to produce genetically-engineered mice to study processes that control cardiorespiratory functions^37,67–69,86–89^. In addition, the C57BL6 mouse strain is heavily used to investigate the underlying processes of sleep apnea because it has irregular breathing patterns, such as spontaneous pauses (i.e., apneas), and type 1 and type 2 sighs during both sleep and wakefulness (including when the mouse is awake and still), and disordered breathing on return to room-air after either hypercapnic or hypoxic gas exposure^73,90–97^. Thus, there is considerable information about the genetic^86,90,92,96–101^ and neurochemical^86,91,102–107^ factors that drive breathing patterns of C57BL6 mice and their responses to HCC. Moreover, the importance of key structural differences in the carotid bodies as they relate to the breathing patterns of C57BL6 mice compared to other strains has also been studied^108–110^.

The roles of endothelial nitric oxide synthase (eNOS) and neuronal nitric oxide synthase (nNOS) in the expression of ventilatory responses to HXC and HCC, and those that occur upon return to room-air in anesthetized and awake mice have received much attention^71,84,85,111,112^. Conclusively, the experiments employed both male and female nNOS-/- and eNOS-/- mice derived from hybrids of C57BL6 and 129/SV mouse strains, and as WT controls for these experiments, individual eNOS and nNOS positive male and female mice from hybrids of C57BL6 and 129/SV strains were used. With respect to responses elicited by HCC in freely-moving mice, the ventilatory responses for the nNOS-/- and eNOS-/- mice were similar to those of the WT mice^71,111,112^. Moreover, the short-term potentiation (STP) of ventilatory parameters after HCC was virtually absent in nNOS-/- mice^84,85^. Kline and colleagues did not report whether eNOS-/- mice were studied or whether the post-HCC responses were different in eNOS-/- or nNOS-/- male or female mice. In addition to the experiments mentioned above, eNOS-/- mice of hybrid 129/SV and C57BL6 background have been used extensively to study the physiological roles of this NOS isoform^74,113–126^.

The primary goal of the present study was to compare the ventilatory responses during and after a 15 min HCC (5% CO_2_, 21% O_2_, 74% N_2_) in adult male and female C57BL6 mice and in eNOS-/- mice of C57BL6 background. Our data shows that (1) the ventilatory responses during and after HCC were similar in male and female C57BL6 WT mice; (2) the responses during and after HCC in male eNOS-/- mice were substantially smaller than in male WT mice; and (3) the responses in female eNOS-/- mice during and after HCC were similar to those in female WT mice. Our studies complement those of Kline and colleagues^71,84,85,111,112^, and strongly suggest that sex and genetics play a major role in the importance of eNOS in the ventilatory responses to HCC.

## Methods

### Permissions

All the studies described in this manuscript received prior approval of the Animal Care and Use Committees of the University of Virginia and Case Western Reserve University. All the surgical procedures, post-surgical handing of the mice and experimental protocols were performed in strict accordance with the National Institutes of Health (NIH) guidelines for care and use of laboratory animals (https://www.nap.edu/catalog/5140/guide-for-the-care-and-use-of-laboratory-animals). In addition, all studies were carried out in strict compliance with the ARRIVE (Animal Research: Reporting of In Vivo Experiments) guidelines (http://www.nc3rs.org.uk/page.asp?id=1357).

### Mice

Adult male and female homozygous eNOS-/- mice (C57BL6J-NOS3tm1Unc) with C57BL6J (C57BL6) genetic background and age-matched male and female C57BL6 mice were purchased from Jackson Laboratory (Bar Harbor, ME, USA). Mice were delivered pathogen free and housed under specific-pathogen free conditions with a 12 h light–dark cycle. The study did not take into account the status of the menstrual cycle of the female mice because of evidence that (1) the menstrual cycle has minor effects on breathing parameters and ventilatory responses to HCC, (2) the responsiveness during the luteal phase is greater by only 5–10% than during the follicular phase^127,128^, and (3) there is no observable correlation between the HCC response and the ratio of progesterone and estradiol^129^.

### Whole body plethysmography

All plethysmography studies were done in a quiet laboratory with room temperature of 21.1 ± 0.3 °C and relative humidity of 50.1 ± 0.9%. Each freely-moving mouse was placed in an individual whole body plethysmograph (Buxco Small Animal Whole Body Plethysmography, DSI a division of Harvard Biosciences, Inc., St. Paul, MN, USA) to continuously record ventilatory parameters (Table 1) as detailed previously^37,62–69,73^. The plethysmography system uses a solid state differential pressure transducer, which measures the differential pressure across a Lilly (screen) pneumotachometer, and chamber temperature and relative humidity are continuously monitored by built-in sensors. Fine Pointe software (Buxco) includes single values for body temperature that are set at 36.6 °C and 36.7 °C, respectively, and this software constantly corrected digitized values for fluctuations in both chamber temperature and humidity so that the values do not change during the recording protocol. The complete details of how the plethysmography system measures ventilatory parameters and the technical description of all software and hardware, including the sensors, can be found at this website: https://support.datasci.com/hc/en-us/articles/360006766274-Buxco-Bias-Flow-Manual.

**Table 1:** List of abbreviations.

Category	Abbreviation	Description
General	eNOS	Endothelial nitric oxide synthase
	eNOS-/-	Endothelial nitric oxide synthase knock-out mice
	nNOS	Neuronal nitric oxide synthase
	WT mice	Wild-type mice
	HCC	Hypercapnic gas challenge
	HXC	Hypoxic gas challenge
	RA	Room-air
Ventilatory parameters	Freq	Frequency of breathing
	TV	Tidal volume
	MV	Minute ventilation
	Ti	Inspiratory time
	Te	Expiratory time
	PIF	Peak Inspiratory Flow
	PEF	Peak Expiratory Flow
	Inspiratory drive	Tidal volume/Inspiratory time (TV/Ti)
	Expiratory drive	Tidal volume/Expiratory time (TV/Te)

The array of ventilatory parameters chosen provide a full characterization of the differences in resting breathing patterns and responses to the HCC^37,62–69,73,102,130,131^. The recorded parameters were (a) frequency of breathing (Freq), tidal volume (TV), inspiratory time (Ti, duration of inspiration) and expiratory time (Te, duration of expiration), and peak inspiratory flow (PIF) and peak expiratory flow (PEF). Derived parameters included minute ventilation (MV, Freq x TV), and inspiratory drive (TV/Ti) and expiratory drive (TV/Te). The derived ventilatory parameter inspiratory drive (TV/Ti) is an index of the intensity of the output of brainstem medullary inspiratory neuronal circuits and mechanical output of the respiratory muscles, such as those in the diaphragm and external intercostal muscles^37,44,67–69,73^. This drive (also known as inspiratory effort) is tonically active and is modulated by changes in carotid body chemoafferent input and CO_2_/H^+^-activation of central chemoreceptors in the RTN^37,44,67–69,73^. In addition, the derived ventilatory parameter expiratory drive (TV/Te) is an index of the intensity of the output of the brainstem medullary neuronal circuits and mechanical output of the diaphragm and internal intercostal muscles. This drive (also known as expiratory effort) is not tonically active (i.e., expiration normally occurs passively), but switches to an active/dynamic state via CB afferent input and CO_2_/H^+^-activation of central chemoreceptors within the RTN^37,44,67–69^.

As detailed at https://support.datasci.com/hc/en-us/articles/360006766274-Buxco-Bias-Flow-Manual, respiratory waveforms were continuously produced by converting chamber box flows using built-in commercial software (Buxco Research Systems, Biosystem XA software) that utilized the primary algorithm of Epstein and Epstein^132^. Pressure changes associated with the respiratory waveforms were converted to volumes (e.g., TV, PIF and PEF) also using the algorithm of Epstein and Epstein^132^. The cycle analyzers filtered the acquired signals, factoring in chamber temperature and humidity, and proprietary algorithms (Fine Pointe, Buxco) generated box flow data that identified a breath. From this data, the minimum and maximum box flow values were determined. Minimum and maximum box flows were then multiplied by the compensation factor provided by the selected algorithm, thus providing TV, PIF and PEF values. A rejection algorithm analyzing the breath-by-breath recordings excluded nasal breathing events (i.e., sniffs). Sniffing events are detected by the Fine Pointe software and are readily determined to be too rapid and too shallow to be tidal volume breaths and therefore are rejected by the embedded software. These are rare events once the mice have acclimatized to the chambers and ceased the urge to smell/sniff their new chamber environment^73^.

Calibration of the system is performed by injecting a known volume of air (e.g., 1 ml for mouse chambers) into the plethysmograph and measuring the pressure signal generated by the injection. By integrating the pressure signal with respect to time, a calibration factor is derived which relates pressure change to flow in order to establish analogue gain and offset parameters. The specific steps include: (1) balance the instrumentation amplifier with no signal applied (AMP balance), (2) zero/tare the output of the amplifier using the fine balance controller to establish the zero-flow reference, (3) inject the known volume of air into the plethysmography chamber and measure the pressure generated from the flow, (4) integrate the signal with respect to time to establish the calibration factor, (5) ensure the effective range of the instrument is within the expected flow range of the animal, (6) adjust the gain as necessary to establish an acceptable input range, and (7) repeat steps 2–6 if the gain was adjusted.

### Hypercapnic gas challenge

WT (C57BL6) and eNOS-/- mice were placed in individual plethysmography chambers and allowed 45–60 min to acclimatize to the chambers. In agreement with our previous studies^37,67–69,73^, the female and male C57BL6 mice generally settled within 30–45 min (i.e., the mice were usually still, although they occasionally groomed or moved about the chamber) and so the final 15 min before the gas challenge generally provide stable baseline values or pre-values. An occasional female or male mouse would not settle and thus was not included in the study. After the acclimatization period, the mice were then exposed to a hypercapnic-normoxic gas challenge (HCC, 5% CO_2_, 21%O_2_, 76% N_2_) for 15 min after which time room-air was reintroduced for 15 min. The average temperature of the chambers prior to HCC was 21.5 ± 0.2 °C (*P* > 0.05 chamber pre-value *versus* room-air) and was 21.2 ± 0.4 °C after 15 min of HCC (*P* > 0.05, 15 min HCC value *versus* chamber pre-value).

### Body temperature recordings during and after HCC

All body temperature studies were done in a quiet laboratory with room temperature of 21.2 ± 0.2 °C. To determine the effects of HCC on colonic body temperature we used (a) female C57BL6 mice (n = 6, 85.7 ± 0.9 days of age, 19.4 ± 0.3 g body weight), (b) female eNOS-/- (n = 6, 85.3 ± 0.8 days of age, 18.9 ± 0.3 g body weight), (c) male C57BL6 mice (n = 6, 85.0 ± 0.4 days of age, 25.1 ± 0.3 g body weight), and (d) male eNOS-/- (n = 6, 84.5 ± 0.6 days of age, 24.6 ± 0.2 g body weight). The body weights of female C57BL6 and eNOS-/- mice were similar to one another (*P* > 0.05). The body weights of male C57BL6 and eNOS-/- mice were also similar to one another (*P* > 0.05).

The mice, thermistor probe (YS 451, Yellow Springs Instruments, Yellow Springs, OH), and connected battery-operated thermometer unit (YSI 400) that was used to monitor and record body temperature, were placed in a Hypercapnic Work Station (Coy, Grass Lake, MI), which had room-air flowing through the housing chamber. This chamber allowed access to the mice and recording equipment via flexible air-tight armholes. After 5 min, the thermistor probe was inserted 1.5–2.0 cm rectally in each mouse to record colonic body temperature. The mice were removed and a hypercapnic environment (5% CO_2_, 21% O_2_, 76% N_2_) was established in the chamber using a Pro:Ox : Model 350 unit (Biospherix, Lacona, NY). The mice were then put back into the chamber when the hypercapnic environment was established and body temperature was recorded at 5 and 15 min. After the 15-min recording, the chamber door was opened allowing the inside of the chamber to equilibrate to a room-air environment, and body temperature was again recorded at 5 and 15 min time intervals. Note, that these male and female WT and eNOS-/-mice were different from those that were used for the plethysmography studies described above. Nonetheless, the body temperatures were taken during the same hypercapnic and room-air environments as the plethysmography studies. As seen below in Table 3, the changes in body temperature in every group were less than 0.1 °C during HCC and upon return to room-air and therefore would not impact the accuracy of the ventilatory recordings.

### Statistics

To determine the total responses during HCC and return to room-air, we summed (1) the 20 data points (1 data point every 15 s, therefore 4 data points per 1 min and 20 data points per 5 min) for each mouse recorded over the 5 min immediately before HCC (i.e., pre-values), (2) the 60 data points recorded during the HCC (4 data points per 1 min and 60 data points per 15 min), and (3) the 60 data points recorded following return to room-air (4 data points per 1 min, therefore 20 data points for the first 5 min of return to room-air, and 60 data points for the entire 15 min return to room-air). Cumulative response during the 15 min HCC was calculated as the sum of the 60 data points during HCC—(sum of the 20 data points before HCC (i.e., pre-values) × 3). The cumulative response for the first 5 min of the post-HCC phase or room-air phase was determined as the sum of 20 data points during the 5 min post-HCC phase—(sum of the 20 data points before HCC (i.e., pre-values)). The cumulative response for the 15 min post-HCC phase was determined as the sum of the 60 data points during the post-HCC phase—(sum of the 20 data points before HCC (i.e., pre-values) × 3). The mean and SEM of the group data was then determined.

The baseline value used to calculate the delta responses recorded over the first 90 s of the HCC in each mouse was the average of the values recorded prior to the HCC (i.e., pre-values). The baseline value used to calculate the delta responses recorded over the first 90 s upon return to room-air in each mouse was also the average of the values recorded prior to the HCC (i.e., pre-values). As such, these delta changes recorded over the first 90 s upon return to room-air do not provide the exact delta change for the initial transient spike that occurred upon return to room-air (i.e., delta change from the last HCC value immediately before switching to room-air exposure). However, it should be noted that this way of analysis does show that the large majority of responses during the transient spike were similar between the male WT and eNOS KO mice and between the female WT and eNOS KO mice.

**Figure 1:**
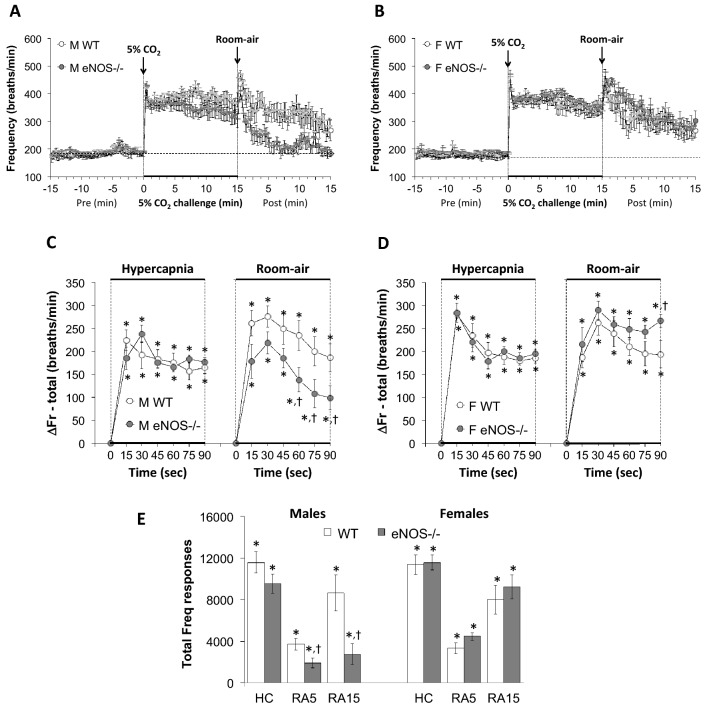
Panels (**A**) and (**B**) Frequency of breathing (Freq) values before, during a hypercapnic (HC) gas challenge (5% CO_2_, 21% O_2_, 74% N_2_) and upon return to room-air in male (M) and female (F) wild-type (WT) and eNOS knock-out (eNOS-/-) mice. Panels (**C**) and (**D**) Arithmetic changes in Freq in male and female WT and eNOS-/- mice during the first 90 s of exposure to HC challenge and the first 90 s upon return to room-air. Panel (**E**) Total changes in Freq in male and female WT and eNOS-/- mice during HC challenge and during the first 5 min (RA5) and entire 15 min (RA15) return to room-air. The data are presented as mean ± SEM. *P < 0.05, significant change from pre-values. ^†^*P* < 0.05, eNOS-/- *versus* WT within each sex.

**Figure 2:**
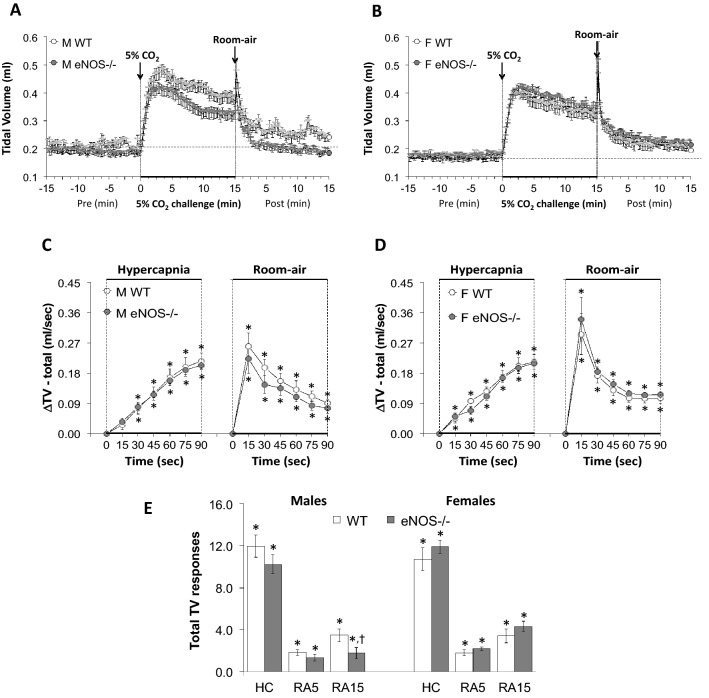
Panels (**A**) and (**B**): Tidal Volume (TV) values before, during a hypercapnic (HC) gas challenge (5% CO_2_, 21% O_2_, 74% N_2_) and upon return to room-air in male (M) and female (F) wild-type (WT) and eNOS knock-out (eNOS-/-) mice. Panels (**C**) and (**D**) Arithmetic changes in TV in male and female WT and eNOS-/- mice during the first 90 s of exposure to the HC challenge and the first 90 s upon return to room-air. Panel (**E**) Total changes in TV in male and female WT and eNOS-/- mice during the HC challenge and during the first 5 min (RA5) and entire 15 min (RA15) return to room-air. The data are presented as mean ± SEM. *P < 0.05, significant change from pre-values. ^**†**^*P* < 0.05, eNOS-/- *versus* WT within each sex.

**Figure 3:**
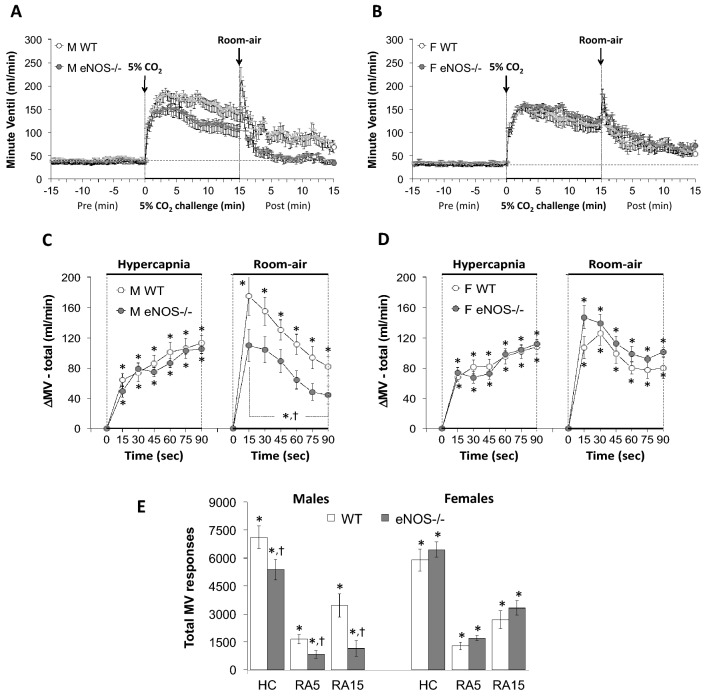
Panels (**A**) and (**B**) Minute Ventilation (MV) values before, during a hypercapnic (HC) gas challenge (5% CO_2_, 21% O_2_, 74% N_2_) and upon return to room-air in male (M) and female (F) wild-type (WT) and eNOS knock-out (eNOS-/-) mice. Panels (**C**) and (**D**) Arithmetic changes in MV in male and female WT and eNOS-/- mice during the first 90 s of exposure to the HC challenge and the first 90 s upon return to room-air. Panel (**E**) Total changes in MV in male and female WT and eNOS-/- mice during the HC challenge and during the first 5 min (RA5) and entire 15 min (RA15) return to room-air. The data are presented as mean ± SEM. *P < 0.05, significant change from pre-values. ^**†**^*P* < 0.05, eNOS-/- *versus* WT within each sex.

**Figure 4:**
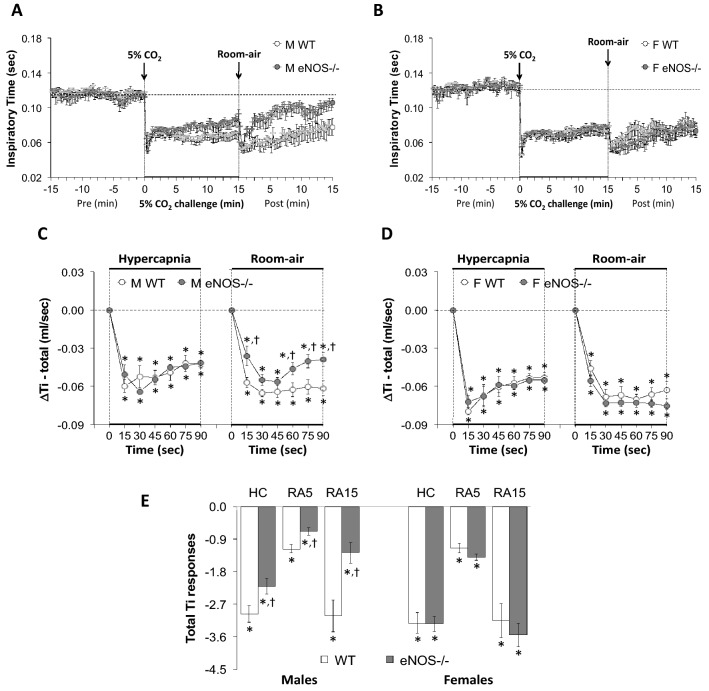
Panels (**A**) and (**B**) Inspiratory time (Ti) values before, during a hypercapnic (HC) gas challenge (5% CO_2_, 21% O_2_, 74% N_2_) and upon return to room-air in male (M) and female (F) wild-type (WT) and eNOS knock-out (eNOS-/-) mice. Panels (**C**) and (**D**) Arithmetic changes in Ti in male and female WT and eNOS-/- mice during the first 90 s of exposure to the HC challenge and the first 90 s upon return to room-air. Panel (**E**) Total changes in Ti in male and female WT and eNOS-/- mice during HC challenge and during the first 5 min (RA5) and entire 15 min (RA15) return to room-air. The data are presented as mean ± SEM. *P < 0.05, significant change from pre-values. ^**†**^*P* < 0.05, eNOS-/- *versus* WT within each sex.

**Figure 5:**
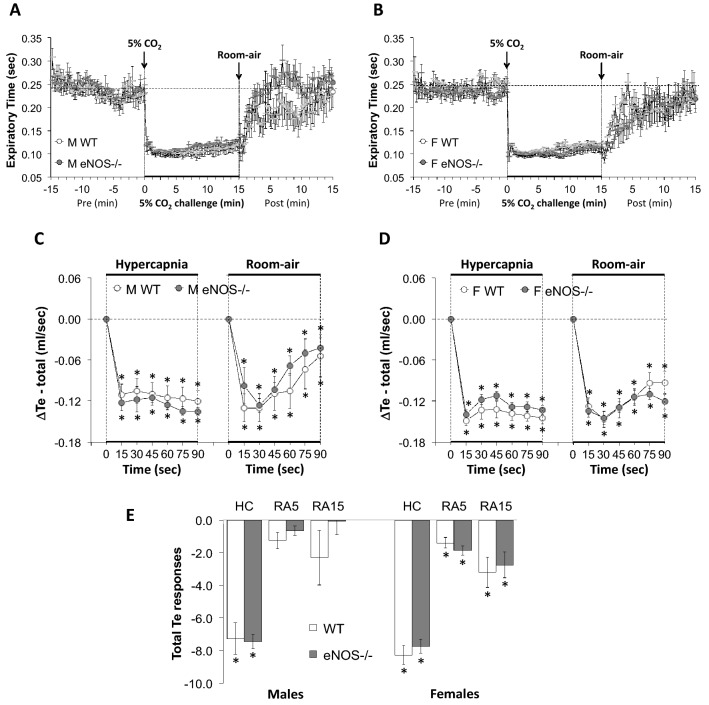
Panels (**A**) and (**B**) Expiratory time (Te) values before, during a hypercapnic (HC) gas challenge (5% CO_2_, 21% O_2_, 74% N_2_) and upon return to room-air in male (M) and female (F) wild-type (WT) and eNOS knock-out (eNOS-/-) mice. Panels (**C**) and (**D**) Arithmetic changes in Te in male and female WT and eNOS-/- mice during the first 90 s of exposure to HC challenge and the first 90 s upon return to room-air. Panel (**E**) Total changes in Te in male and female WT and eNOS-/- mice during the HC challenge and during the first 5 min (RA5) and entire 15 min (RA15) return to room-air. The data are presented as mean ± SEM. *P < 0.05, significant change from pre-values. ^**†**^*P* < 0.05, eNOS-/- *versus* WT within each sex.

**Figure 6:**
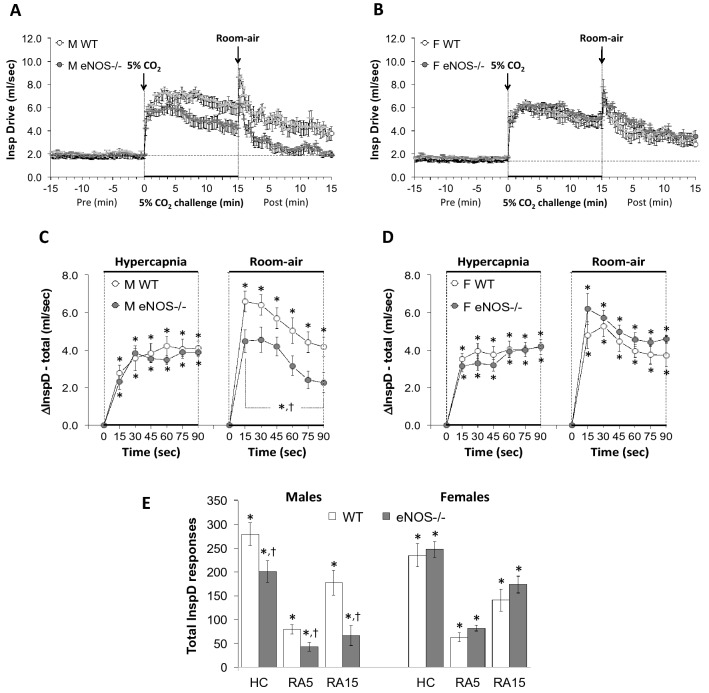
Panels (**A**) and (**B**) Inspiratory drive (tidal volume/inspiratory time, TV/Ti) values before, during a hypercapnic (HC) gas challenge (5% CO_2_, 21% O_2_, 74% N_2_) and upon return to room-air in male (M) and female (F) wild-type (WT) and eNOS knock-out (eNOS-/-) mice. Panels (**C**) and (**D**) Arithmetic changes in TV/Ti in male and female WT and eNOS-/- mice during the first 90 s of exposure to HC challenge and the first 90 s upon return to room-air. Panel (**E**) Total changes in TV/Ti in male and female WT and eNOS-/- mice during HC challenge and during the first 5 min (RA5) and entire 15 min (RA15) return to room-air. The data are presented as mean ± SEM. *P < 0.05, significant change from pre-values. ^**†**^*P* < 0.05, eNOS-/- *versus* WT within each sex.

**Figure 7:**
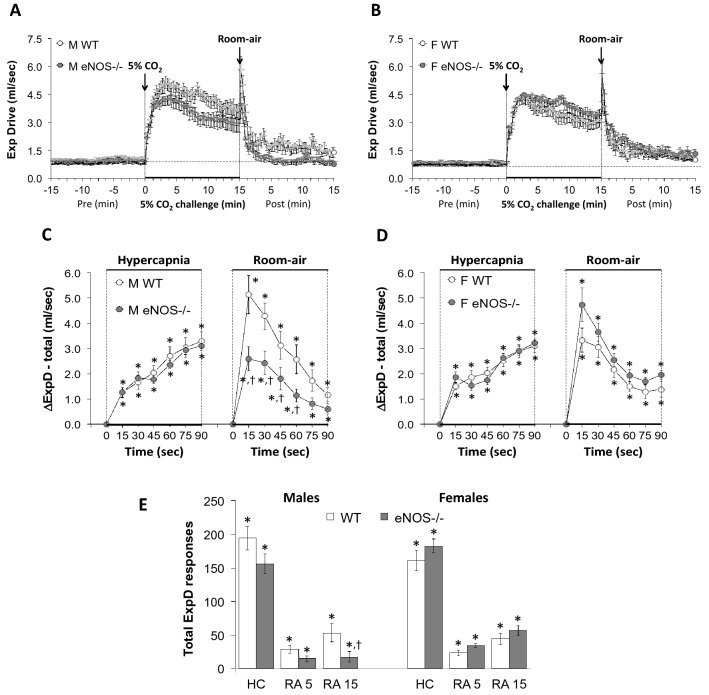
Panels (**A**) and (**B**) Expiratory drive (tidal volume/expiratory time, TV/Te) values before, during a hypercapnic (HC) gas challenge (5% CO_2_, 21% O_2_, 74% N_2_) and upon return to room-air in male (M) and female (F) wild-type (WT) and eNOS knock-out (eNOS-/-) mice. Panels (**C**) and (**D**) Arithmetic changes in TV/Te in male and female WT and eNOS-/- mice during the first 90 s of exposure to HC challenge and the first 90 s upon return to room-air. Panel (**E**) Total changes in TV/Te in male and female WT and eNOS-/- mice during HC challenge and during the first 5 min (RA5) and entire 15 min (RA15) return to room-air. The data are presented as mean ± SEM. *P < 0.05, significant change from pre-values. ^**†**^*P* < 0.05, eNOS-/- *versus* WT within each sex.

**Figure 8:**
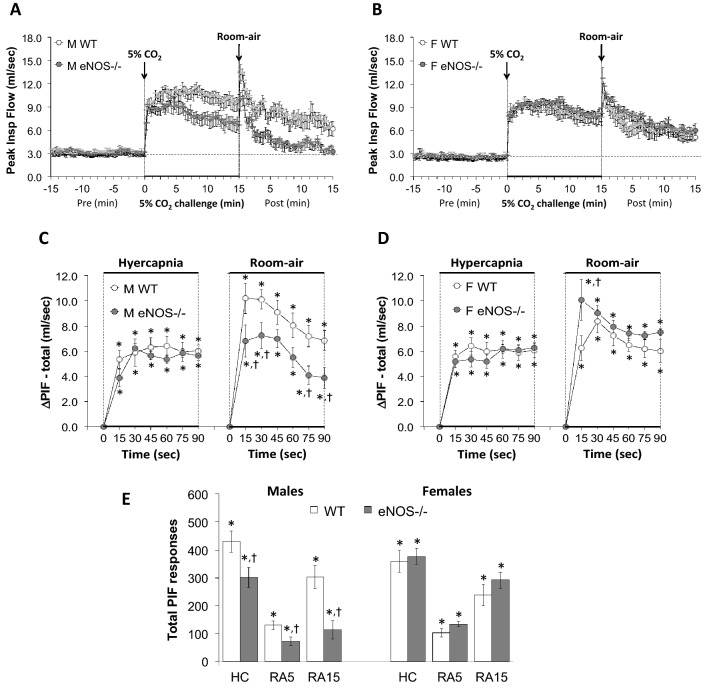
Panels (**A**) and (**B**) Peak Inspiratory Flow (PIF) values before, during a hypercapnic (HC) gas challenge (5% CO_2_, 21% O_2_, 74% N_2_) and upon return to room-air in male (M) and female (F) wild-type (WT) and eNOS knock-out (eNOS-/-) mice. Panels (**C**) and (**D**) Arithmetic changes in PIF in male and female WT and eNOS-/- mice during the first 90 s of exposure to HC challenge and the first 90 s upon return to room-air. Panel (**E**) Total changes in PIF in male and female WT and eNOS-/- mice during HC challenge and during the first 5 min (RA5) and entire 15 min (RA15) return to room-air. The data are presented as mean ± SEM. *P < 0.05, significant change from pre-values. ^**†**^*P* < 0.05, eNOS-/- *versus* WT within each sex.

**Figure 9:**
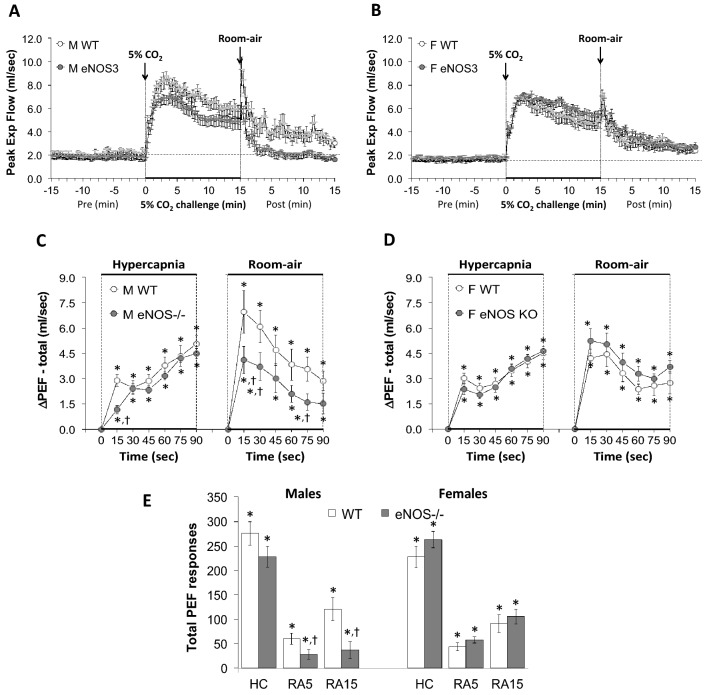
Panels (**A**) and (**B**) Peak Expiratory Flow (PEF) values before, during hypercapnic (HC) gas challenge (5% CO_2_, 21% O_2_, 74% N_2_) and upon return to room-air in male (M) and female (F) wild-type (WT) and eNOS knock-out (eNOS-/-) mice. Panels (**C**) and (**D**) Arithmetic changes in PEF in male and female WT and eNOS-/- mice during the first 90 s of exposure to HC challenge and the first 90 s upon return to room-air. Panel (**E**) Total changes in PEF in male and female WT and eNOS-/- mice during HC challenge and during the first 5 min (RA5) and entire 15 min (RA15) return to room-air. The data are presented as mean ± SEM. *P < 0.05, significant change from pre-values. ^**†**^*P* < 0.05, eNOS-/- *versus* WT within each sex.

All data were analyzed by one-way or two-way ANOVA followed by Student's modified *t*-test with Bonferroni corrections for multiple comparisons between means employing the error mean square terms from each ANOVA^133,134^. An initial value of *P* < 0.05 was taken as the level of statistical significance and modified according to the numbers of between group comparisons^133^. All of the recorded and calculated parameters are presented as mean ± standard error of the mean (SEM). We adhere to the principals outlined by Winer^134^ and Cumming et al^135^ about the presentation of repeated-measures data with SEM rather than standard deviation (SD). SEM quantifies uncertainty in estimating the mean with less uncertainty when larger numbers in the groups are present (as is the case in our data), whereas SD indicates dispersion of the data from the mean value. Inherent variability of data at the beginning can be either (1) carried through the repeated measures, such as in the present study where there are multiple time points or (2) altered as the result of an experimental procedure, such as a hypoxic or hypercapnic gas challenge. Note that we performed multivariate ANOVAs that included sex as a variable and found that the data was highly sex-dependent with male, but not female eNOS-/- mice differing from their respective WT control mice (*P* < 0.001). Also note that the analyses of the differences between female WT and female eNOS-/- mice, namely, 11 parameters × 15 between group comparisons (Figs. 1, 2, 3, 4, 5, 6, 7, 8, and 9, panels C, D and E) = 165 comparisons, yielded only 3 significant differences: one for Freq, one for Ti/Te and one for PIF.

## Results

### Descriptors and baseline values for the WT and eNOS-/- male and female mice used in the whole body plethysmography studies

Table 2 shows the ages and body weights of the mice used in the whole body plethysmography studies, and the average resting ventilatory parameters of these mice recorded over the 15 min period immediately before beginning the HCC. With respect to the female mice, there were no between-group differences in the ages, body weights or resting ventilatory parameters between the WT and eNOS-/- mice. With regards to the male mice, (1) the ages and body weights of the WT and eNOS-/- mice were similar to one another, (2) resting TV (but not Freq) was significantly lower in eNOS-/- mice, such that resting MV (Freq x TV) was also lower in the eNOS-/- mice, and (3) resting expiratory drive and PEF values were significantly lower in eNOS-/- mice. These significant differences in the four above mentioned resting values were not seen in our companion study that looks at the ventilatory responses after hypoxic gas challenge in male and female eNOS-/- mice^136^. A comparison of the values in male WT and male eNOS-/- mice from both this and our companion study and an in depth discussion about the findings are present in the Supplementary File (Supplemental Table 1).

**Table 2:** Resting parameters in female and male wild-type and endothelial nitric oxide synthase knock-out mice.

Parameter	Females		Males	
	WT	eNOS-/-	WT	eNOS-/-
Number of mice	12	13	11	13
Age (days)	86.0 ± 0.6	82.7 ± 2.3	85.2 ± 0.4	87.9 ± 2.2
Body weight (gram)	19.3 ± 0.3	18.3 ± 0.6	25.3 ± 0.6	24.2 ± 0.6
Frequency (breaths/min)	175 ± 5	181 ± 5	190 ± 6	185 ± 3
Tidal volume (ml)	0.170 ± 0.007	0.173 ± 0.004	0.214 ± 0.009	0.184 ± 0.005*
Minute ventilation (ml/min)	29.5 ± 1.0	30.9 ± 1.0	39.8 ± 1.7	34.0 ± 1.3*
Inspiratory time (sec)	0.123 ± 0.003	0.124 ± 0.002	0.115 ± 0.003	0.112 ± 0.002
Expiratory time (sec)	0.246 ± 0.007	0.230 ± 0.007	0.224 ± 0.013	0.236 ± 0.007
Inspiratory time/Expiratory time	0.511 ± 0.016	0.552 ± 0.015	0.534 ± 0.024	0.487 ± 0.016
Inspiratory drive (ml/sec)	1.41 ± 0.05	1.42 ± 0.04	1.90 ± 0.08	1.68 ± 0.06
Expiratory drive (ml/sec)	0.70 ± 0.03	0.77 ± 0.03	0.99 ± 0.05	0.80 ± 0.04*
Peak inspiratory flow (PIF) (ml/sec)	2.38 ± 0.06^†^	2.39 ± 0.05	3.03 ± 0.12	2.86 ± 0.10
Peak expiratory flow (PEF) (ml/sec)	1.57 ± 0.06	1.62 ± 0.06	2.17 ± 0.13	1.67 ± 0.08*
PIF/PEF	1.53 ± 0.05	1.49 ± 0.04	1.44 ± 0.08	1.75 ± 0.06

The data are presented as mean ± SEM. WT, wild-type C57BL6 mice. eNOS-/-, endothelial nitric oxide synthase knock-out mice.

*P < 0.05, eNOS-/- versus WT for male and female mice.

### Colonic body temperature responses

Table 3 summarizes the colonic body temperature responses that occurred before, during and following the HCC in male and female WT and eNOS-/- mice. The male and female mice had similar body temperatures. Neither exposure to HCC nor return to room-air significantly affected body temperature in any of these mice. The body temperatures changed by less than 0.1 °C during HCC and upon return to room-air, and as mentioned in the methods section, this shift in body temperature would minimally impact determination of the plethysmography values.

**Table 3:** Changes in body temperature during a hypercapnic gas challenge and upon return to room-air in male and female wild-type and endothelial nitric oxide synthase knock-out mice.

Sex	Group	Pre	Arithmetic changes in body temperature (°C)			
			Hypercapnia		Room-air	
			5 min	15 min	5 min	15 min
Male	WT	36.5 ± 0.1	− 0.07 ± 0.08	− 0.02 ± 0.07	+ 0.07 ± 0.06	+ 0.00 ± 0.07
	eNOS-/-	36.6 ± 0.1	+ 0.05 ± 0.12	− 0.08 ± 0.12	− 0.02 ± 0.08	+ 0.05 ± 0.06
Female	WT	36.7 ± 0.1	− 0.07 ± 0.11	+ 0.08 ± 0.08	− 0.05 ± 0.12	+ 0.02 ± 0.06
	eNOS-/-	36.8 ± 0.1	− 0.03 ± 0.11	+ 0.07 ± 0.09	− 0.03 ± 0.09	− 0.05 ± 0.03

The data are presented as mean ± SEM. WT, wild-type C57BL6 mice. eNOS-/-, endothelial nitric oxide synthase knock-out mice. There were 6 mice in each group. Note, there were no significant changes in body temperature or any between-group differences at any time point (*P* > 0.05, for all comparisons).

### Sex comparisons in ventilatory responses during and following HCC

The following sections will describe the ventilatory responses that occurred during HCC and upon return to room-air in male and female WT mice. It is important to note that the array of ventilatory responses among the male and female WT mice were qualitatively similar to one another, and there were no quantitative differences in the overall responses between WT males and females during HCC. However, the initial increases in MV, expiratory drive, PIF and PEF upon return to room-air in female WT mice were significantly smaller in magnitude than the male WT mice (Figs. 3, 7, 8 and 9).

### Ventilatory responses during and following HCC in female WT and eNOS-/- mice

As can be seen in Figs. 1, 2, 3, 4, 5, 6, 7, 8, and 9, HCC elicited robust changes in ventilatory parameters in female WT mice. These responses included rapid and sustained increases in Freq (associated with rapid and sustained decreases in Ti and Te), TV, MV, inspiratory and expiratory drives, and PIF and PEF. The Ti/Te ratio rose during HCC (Supplemental Fig. 1) for female WT mice because of the relatively greater decrease in Te than Ti (compare Figs. 4 and 5). The PIF/PEF for the female WT mice ratio did not change during HCC (Supplemental Fig. 2) because the increases in PIF and PEF were of similar magnitude (Figs. 8 and 9). The return to room-air in the female WT mice elicited abrupt transient increases in Freq (associated with abrupt decreases in Ti and Te), TV, MV, inspiratory and expiratory drives, and PIF and PEF, which thereafter, gradually returned toward baseline values (Figs. 1, 2, 3, 4, 5, 6, 7, 8, and 9), with the exception that Te returned to baseline much more quickly than Ti (Figs. 4 and 5). Moreover, the Ti/Te ratio fell dramatically upon return to room-air for the female WT mice because of the rapid recovery of Te values (Supplemental Fig. 1). PIF/PEF ratio rose upon to return to room-air for the female WT mice (Supplemental Fig. 2) because PEF recovered toward baseline values somewhat more quickly that PIF (Figs. 8 and 9). A major finding was that the ventilatory responses in the female eNOS-/- mice were virtually identical to those in the female WT mice and no significant differences were found with the statistical analyses presented in Figs. 1, 2, 3, 4, 5, 6, 7, 8, and 9.

### Ventilatory responses during and following HCC in male WT and eNOS-/- mice

As seen in Figs. 1, 2, 3, 4, 5, 6, 7, 8, and 9, the ventilatory responses during HCC and on return to room-air in male WT mice were similar in every respect to what was described above for female WT mice.

### Frequency (Freq), Tidal Volume (TV) and Minute Ventilation (MV)

As seen in Fig. 1, HCC elicited robust increases in Freq in the male and female WT and eNOS-/- mice (Panels A and B). The rate of reaching the initial increases in Freq (Panels C and D) during HCC and the total (arithmetic) responses (Panel E, column HC) were similar in the male WT and eNOS-/- mice and female WT and eNOS-/- mice. Upon return to room-air, Freq returned to baseline more rapidly in eNOS-/- males compared to WT males than eNOS-/- females compared to WT females (Panels A–D) therefore, the total responses over the 5 and 15 min time points were significantly smaller in the eNOS-/- males compared to the WT males (Panel E, columns RA5 and RA15). There was no difference in the total room-air responses in the female WT and eNOS-/- mice (Panel E, columns RA5 and RA15). As seen in Fig. 2, HCC elicited robust increases in TV in the male and female WT and eNOS-/- mice (Panels A and B). The rate of reaching the initial increases in TV (Panels C and D) during HCC and the total (arithmetic) responses (Panel E, column HC) were similar in male WT and eNOS-/- mice and female WT and eNOS-/- mice. Upon return to room-air, TV returned to baseline at an initial similar rate in both male and female eNOS-/- and WT mice (Panels A–D), but thereafter TV values reached resting conditions significantly more quickly in male eNOS-/- mice compared to WT males than female eNOS-/- mice compared to WT females.  Therefore, the total responses over the entire 15 min time period were significantly smaller in the eNOS-/- males than in the WT males (Panel E, column RA15). There was no difference in the total room-air responses in female WT and eNOS-/- mice at both the 5 min or entire 15 min time points (Panel E, columns RA5 and RA15). As seen in Fig. 3, the changes in Freq and TV resulted in HCC-induced increases in MV in male and female WT and eNOS-/- mice (Panels A and B). The rate of reaching the initial increases in MV (Panels C and D) during HCC were similar in male WT and eNOS-/- mice and female WT and eNOS-/- mice. The total (arithmetic) responses (Panel E, column HC) were similar in female WT and eNOS-/- mice, but significantly smaller in male eNOS-/- mice than in male WT mice. Upon return to room-air, MV returned to baseline at an initial faster rate in male eNOS-/- mice than in male WT mice (Panels A and C), and returned to baseline at a similar initial rate in female eNOS-/- and WT mice (Panels B and D). After the initial 90 s, MV values reached baseline values more quickly in male eNOS-/- mice compared to WT males than female eNOS-/- mice compared to WT females, thus the total responses over the 5 min and 15 min periods were significantly smaller in eNOS-/- males than in WT males (Panel E, columns RA5 and RA15). There was no difference in the total room-air responses in the female WT and eNOS-/- mice (Panel E, columns RA5 and RA15).

### Inspiratory time (Ti), Expiratory time (Te) and Ti/Te ratio

As seen in Fig. 4, HCC elicited robust decreases in Ti in the male and female WT and eNOS-/- mice (Panels A and B). The rate of reaching the initial decreases in Ti during HCC were similar in male WT and eNOS-/- mice and female WT and eNOS-/- mice (Panels C and D). The total (arithmetic) responses (Panel E, column HC) were similar in female WT and eNOS-/- mice, but significantly less in male eNOS-/- mice than in WT males. Upon return to room-air, Ti returned to baseline more rapidly in eNOS-/- males compared to WT males than eNOS-/- females compared to WT females (Panels A–D). Therefore, the total responses over the 5 and 15 min periods were significantly smaller in eNOS-/- males than in WT males (Panel E, columns RA5 and RA15). There was no difference in the total room-air responses in the female WT and eNOS-/- mice (Panel E, columns RA5 and RA15).

As seen in Fig. 5, HCC elicited robust decreases in Te (Panels A and B) in male and female WT and eNOS-/- mice. The rate of reaching the initial decreases in Te (Panels C and D) during HCC and the total (arithmetic) responses (Panel E, column HC) were similar in male WT and eNOS-/- mice and female WT and eNOS-/- mice. Upon return to room-air, Te returned to baseline similarly in WT and eNOS-/- males and WT and eNOS-/- females (Panels A–D) such that the total responses over the 5 and 15 min periods were not statistically different in either groups of mice (Panel E, columns RA5 and RA15). As seen in Supplemental Fig. 1, the Ti/Te ratio rose equally in male and female WT and eNOS-/- mice (due to a relatively greater shortening of Te than Ti) during the HCC, whereas Ti/Te fell below baseline in both the male and female WT and eNOS-/- mice upon return to room-air (due to the more rapid return of Te toward baseline). We calculated the Ti/Te ratio in order to highlight the differential effects of respiratory timing during HCC and the return to room-air, and thus we can conclude that loss of eNOS-/- is not significant for respiratory timing.

### Inspiratory drive (TV/Ti) and Expiratory drive (TV/Te)

As seen in Fig. 6, HCC elicited robust increases in inspiratory drive (TV/Ti) in male and female WT and eNOS-/- mice (Panels A and B). The rate of reaching the initial increases in TV/Ti (Panels C and D) during HCC were similar in WT and eNOS-/- males and WT and eNOS-/- females. The total (arithmetic) responses (Panel E, column HC) were similar in WT and eNOS-/- females, but significantly less in male eNOS-/- mice than WT males. This was due to less of a sustained increase in TV with a smaller decrease in Ti in eNOS-/- male mice compared to WT male mice (Figs. 2 and 4, respectively). Upon return to room-air, TV/Ti returned to baseline at an initial faster rate in male eNOS-/- mice than in male WT mice (Panels A and C) and returned to baseline at a similar initial rate in eNOS-/- and WT female mice (Panels B and D). After the initial 90 s, TV/Ti values recovered to baseline values more quickly in male eNOS-/- mice compared to WT males, than female eNOS-/- mice compared to WT females, therefore the total responses during the 5 min and 15 min time periods were significantly smaller in eNOS-/- males than WT males (Panel E, columns RA5 and RA15). There was no difference in total room-air responses in WT and eNOS-/- female mice (Panel E, columns RA5 and RA15). As seen in Fig. 7, HCC elicited robust increases in expiratory drive (TV/Te) in male and female WT and eNOS-/- mice (Panels A and B). The rates of reaching the initial increases in TV/Te during HCC were similar in male WT and eNOS-/- mice and female WT and eNOS-/- mice (Panels C and D). The total (arithmetic) responses (Panel E, column HC) were similar in WT and eNOS-/- male and female mice. Upon return to room-air, TV/Te values returned to baseline values at an initial faster rate in eNOS-/- males than in WT males (Panels A and C) and returned to baseline values at a similar initial rate in WT and eNOS-/- female mice (Panels B and D). After the initial 90 s, TV/Te values reached baseline values more quickly in male than female eNOS-/- mice, such that the total responses over the 15 min period were significantly smaller in eNOS-/- males than in WT males (Panel E, column RA15). There was no difference in total room-air responses in female WT and eNOS-/- mice (Panel E, columns RA5 and RA15).

### Peak inspiratory flow (PIF) and peak expiratory flow (PEF)

As seen in Fig. 8, HCC elicited robust increases in PIF in male and female WT and eNOS-/- mice (Panels A and B). The rate of reaching the initial increases in PIF during HCC were similar in both groups (Panels C and D). The total (arithmetic) responses (Panel E, column HC) were similar in female WT and eNOS-/- mice, but significantly less in the male eNOS-/- mice compared to the WT males. Upon return to room-air, PIF returned to baseline at an initial faster rate in male eNOS-/- mice than male WT mice (Panels A and C) and returned to baseline at a similar initial rate in eNOS-/- and WT female mice (Panels B and D). After the initial 90 s, PIF values reached baseline values more quickly in eNOS-/- males compared to WT males than eNOS-/- females compared to WT females, therefore the total responses over the 5 min and 15 min periods were significantly smaller in the eNOS-/- males than the WT males (Panel E, columns RA5 and RA15). There was no difference in the room-air total responses in female WT and eNOS-/- mice (Panel E, columns RA5 and RA15). As seen in Fig. 9, HCC elicited robust increases in PEF in male and female WT and eNOS-/- mice (Panels A and B). The rate of reaching the initial increases in PEF (Panels C and D) during HCC were similar in both groups. The total (arithmetic) responses (Panel E, column HC) were similar in WT and eNOS-/- females, but significantly less in the male eNOS-/- mice than male WT mice. Upon return to room-air, PEF returned to baseline values at an initial faster rate in male eNOS-/- mice compared to the male WT mice (Panels A and C) and returned to baseline at a similar initial rate in female eNOS-/- and WT mice (Panels B and D). After the initial 90 s, PEF values reached baseline values more quickly in male eNOS-/- mice compared to WT males than female eNOS-/- mice compared to WT females, therefore the total responses over the 5 min and 15 min periods were significantly smaller in eNOS-/- males than in WT males (Panel E, columns RA5 and RA15). There was no difference in total room-air responses in the female WT and eNOS-/- mice (Panel E, columns RA5 and RA15). As seen in Supplemental Fig. 2, HCC elicited an initial increase in PIF/PEF ratio at the 15 s time point that was greater in magnitude in eNOS-/- males than WT males (Panels A and C). There was no initial difference following HCC in the female WT and eNOS-/- mice (Panels B and D). The total (arithmetic) responses during the HCC (Panel E, column HC) of the PIF/PEF ratio fell in the eNOS-/- male mice, but not WT male mice. Moreover, the total responses during the HCC (Panel E, column HC) of the PIF/PEF ratio fell slightly in the eNOS-/- females, but not WT females, however this drop in PIF/PEF ratio did not reach statistical significance.  Upon return to room-air, the PIF/PEF ratio rose to similar levels in both groups at the 5 and 15 min time points,  (Panels A–E) due to a greater relative fall in PIF than PEF (Figs. 8 and 9, respectively).

## Discussion

The present study found a sex difference in the role of eNOS in baseline ventilatory parameters recorded. More specifically, resting values of TV, MV, PEF and expiratory drive were significantly lower in male eNOS-/- C57BL6 mice than in male C57BL6 WT mice, whereas there were no differences in any resting parameter between the female C57BL6 eNOS-/- and WT mice. These findings differ from our companion study, in which we found that resting ventilatory parameters in male and female C57BL6 eNOS-/- mice were similar to their respective male and female C57BL6 WT controls^136^. The ages and weights of the mice in the present study and that of Getsy et al^136^ were similar to one another, and all mice were fed identical diets and from the same vendor, Jackson Laboratory (Bar Harbor, ME, USA). In addition, both sets of studies were performed between 10 am and noon and were done between the middle of October and early January the following year. As such, we do not have a definitive explanation for why male eNOS-/- mice in the present study have lower resting TV, MV, PEF and expiratory drive than the WT male mice. Nonetheless, since it is only a select few of the baseline parameters that differ in male eNOS-/- and WT mice, it appears that eNOS is not critically essential for maintenance of baseline respiratory timing (e.g., Freq, Ti, Te, EIP and EEP), ventilatory mechanics (e.g., TV, PIF and PEF), and the products of timing and mechanics, including MV and inspiratory and expiratory drives in either sex. Our baseline findings pertaining to eNOS-/- mice of C57BL6 background are in general agreement with those of Kline et al^112^ who reported that resting Freq, TV and MV in eNOS-/- mice were similar to WT controls, which consisted of hybrids of 129/SV and C57BL6 strains of mice.

With respect to the differences in baseline values found in this study, but not in our companion study^136^, it should be noted that when we pooled the baseline data of the male WT and eNOS-/- mice from the present (hypercapnia) study with those of the male WT and eNOS-/- mice from our published hypoxia study^136^, there are no statistical differences in TV, MV, expiratory drive and PEF between the male WT and eNOS-/- mice. This suggests that sampling of a wider population of male WT and male eNOS-/- would likely show that there are no differences in resting ventilatory parameters between these two groups. Nonetheless, we felt it imperative to discuss the between-group differences in baseline TV, MV, expiratory drive and PEF that were identified in the present study.

The colonic body temperature of the WT male and female C57BL6 mice were similar to one another prior to the HCC. This is an agreement with evidence that body temperatures are similar in male and female C57BL6 mice^137^, although there is evidence that body temperatures of female C57BL6 mice can be approximately 0.5 °C higher than the males^138^. In agreement with previous findings from other laboratories^139,140^, the body temperature of the male eNOS-/- mice were similar to those of male WT mice. Additionally, the body temperatures of the female eNOS-/- mice were similar to those of the female WT mice, although no published studies exist pertaining to female WT and eNOS-/- mice. Moreover, body temperatures did not change during or following HCC in the female or male eNOS-/- or WT mice, suggesting that C57BL6 mouse strain can readily maintain thermoregulatory status in the absence of eNOS.

Exposure to HCC elicited an array of responses in the male and female WT mice, including (1) abrupt and sustained increases in Freq that were accompanied by equally abrupt and sustained decreases in Ti and Te (the relative decreases in Te were larger than the decreases in Ti, resulting in sustained increases in the Ti/Te ratio during HCC), and (2) abrupt increases in TV, MV, PIF, PEF, inspiratory drive and expiratory drive. The increases in TV, MV, PEF and expiratory drive were subject to mild roll-off, whereas the increases in PIF and inspiratory drive were not. The responses in the WT female mice were similar in magnitude and duration to those in male WT mice. Additionally, the PIF/PEF ratios did not change from baseline in either sex since the increases in PIF and PEF were similar in magnitude in male and female WT mice. Our study supports previous studies demonstrating that HCC is a powerful respiratory stimulant in mice^112,131–143^, and extend these findings to unreported parameters including PIF, PEF and inspiratory drive and expiratory drive.

An important finding of our study was that the total increase in MV in male eNOS-/- mice during the HCC was significantly smaller than in male WT mice. This was somewhat unexpected since the total Freq and TV responses were not significantly smaller in male eNOS-/- mice than WT male mice during HCC. Nonetheless, the combined changes (Freq x TV) resulted in a decreased total MV in the eNOS-/- male mice compared to the male WT mice. The increase in Freq during HCC in the male eNOS-/- mice trended to be, but was not significantly less than in the WT mice. The total fall in Ti, but not Te, was significantly less during HCC in the male eNOS-/- than in the male WT mice. The total increases in PIF and inspiratory drive were also significantly smaller in male eNOS-/- mice than the male WT mice, whereas the increases in PEF and expiratory drive were similar in eNOS-/- and WT male mice. As such, it appears that the loss of eNOS in male C57BL6 mice has detrimental effects on inspiratory responses, but not expiratory responses to HCC.

Moreover, a major finding was that the post-hypercapnic STP of ventilation responses that occurred upon return to room-air, namely increases in Freq, TV, MV, PIF, PEF, inspiratory drive and expiratory drive, along with decreases in Ti and Te, were markedly diminished in male eNOS-/- mice compared to male WT mice. This suggests that the ventilatory responses in male eNOS-/- mice simply involved a rapid return to baseline values after HCC, whereas in male WT mice, STP of ventilation often lasted for 10–15 min after HCC.

It is not known the mechanisms by which the absence of eNOS contributes to the observed differences between male WT and male eNOS-/- C57BL6 mice in response to HCC and post-hypercapnia room-air exposure, but it is tempting to assume that absence of eNOS within the carotid body-carotid sinus nerve complex, and central structures (e.g., retrotrapezoid nucleus) is involved. Our laboratory recently demonstrated that the initial changes in ventilatory parameters that resulted upon exposure to a HCC (5% CO_2_, 21% O_2_, 74%N_2_) occurred more slowly in male C57BL6 mice with bilateral transection of the carotid sinus nerve than those of sham-operated mice, although similar maximal responses occurred in both groups^163^. In addition, the post-HCC ventilatory responses in mice with bilateral carotid sinus nerve transection were virtually absent, suggesting a vital role of the carotid body in the expression of the post-HCC responses^163^. It therefore appears from our data that the presence (or absence of eNOS) does not contribute to the initiation of the carotid body-dependent responses elicited by HCC in male C57BL6 mice, but that eNOS is a vital contributor to the post-HCC responses in male C57BL6 mice. Whether this involves eNOS at the level of the carotid body and/or brainstem sites, which integrate and process the ventilatory responses to HCC, remains to be determined.

The presence and distribution of eNOS in the carotid body of the mouse (or human) has not yet been reported, but eNOS is found in several structures in the carotid bodies of various species. For instance, in the rat, it is well documented that eNOS is present in (a) nerve terminals of carotid sinus chemoafferents, (b) post-ganglionic sympathetic fibers that emanate from the superior cervical ganglia and innervate vasculature in the carotid body, (c) the vascular endothelium itself^22,144–151^, and (d) carotid body chemosensitive glomus (type I) cells^149,150^. The majority of functional studies have shown that nitric oxide, a product of NOS, has an inhibitory role within the carotid bodies of all species studied^152–159^, although there is evidence that nitric oxide has both an inhibitory and excitatory effect on cat carotid body chemoreception^160,161^. In addition, the generation of S-nitrosothiols (e.g., S-nitrosocysteine and S-nitrosoglutathione), formed via the binding of a sulfur ion to nitric oxide, in the carotid bodies and blood circulation may play a pivotal role in the carotid body-carotid sinus nerve mediated increase in ventilation upon return to room-air in response to HCC. This hypothesis is supported by findings suggesting that (1) the post-hypoxic ventilatory responses are dramatically reduced in carotid sinus nerve transected mice and in mice in which red blood cell hemoglobin cannot generate S-nitrosothiols^37^, (2) the post-hypoxic ventilatory responses are markedly augmented in mice lacking the enzyme that degrades S-nitrosoglutathione^69^, and (3) close arterial injections of S-nitroso-L-cysteine causes a pronounced increase in minute ventilation via activation of carotid sinus nerve chemoafferents through mechanisms involving voltage-gated K^+^-channels^162^. Whether carotid body generated S-nitrosothiols or circulating S-nitrosothiols are involved in the carotid sinus nerve-dependent post-hypercapnic responses remains to be established.

Another key finding was that the HCC and post-HCC responses in female eNOS-/- mice were virtually identical to those of female WT mice for all parameters. These findings clearly suggest the female C57BL6 mice are able to compensate for the loss of eNOS in ways that male C57BL6 mice cannot, at least with respect to the responses during and after a HCC. Our companion manuscript also found that the post-hypoxic responses were diminished in male eNOS-/- mice as compared to male WT C57BL6 mice, whereas the post-hypoxic responses in the female eNOS-/- mice were as robust as those in female WT mice^136^. Therefore, genetics has an important influence on the role of eNOS in the ventilatory responses that occur after hypoxia or hypercapnia gas challenges in C57BL6 mice. Finally, it should be noted that our findings contradict somewhat with those of Kline and his colleagues^71,111,112^. In brief, Kline and his colleagues used adult male and female eNOS-/- mice derived from hybrids of 129/SV and C57BL6 strains of mice and used eNOS intact hybrids as wild-type (WT) controls. With respect to the ventilatory responses elicited by HCC, their studies found that the ventilatory responses during HCC in male and female eNOS-/- mice were similar to those in the male and female WT mice^71,111,112^.

With respect to the physiological basis of the post-HCC responses, it is important to note that the responses for both groups lasted for up to 15 min for most of the recorded ventilatory parameters. The post-HCC ventilatory responses were associated with minimal changes in behavior in that the mice showed no immediate response to the return to room-air and there were no increases in behaviors, such as grooming or moving about the cage. We have determined that transitioning from room-air to a normoxic gas mixture (21% O_2_, 79% N_2_ at 0.5 L/min) from a commercial gas tank and then back to room-air minimally affects ventilatory parameters, such as frequency of breathing in freely-moving male C57BL6 mice (n = 9). The maximal increases in frequency upon introduction of normoxic gas was + 7 ± 5% and the maximal increases in frequency upon reintroduction of room-air was + 8 ± 6% (*P* > 0.05, for both responses). This suggests that the changes in ventilatory parameters upon return to room-air are real changes in ventilatory performance, and not due to changes in activity of the mice or technical issues related to switching of gases.

In summary, our present study demonstrates that eNOS has an essential role in the expression of the ventilatory responses that occur during and after exposure to HCC in adult male C57BL6 mice, whereas the ventilatory responses in adult females are not obviously compromised by the loss of eNOS. Whether redundant systems or expression of inducible proteins/signaling processes allow for full expression of responses in female mice remains to be determined. Why the males are not able to compensate for the loss of eNOS is an obviously intriguing question. The complex interactions of eNOS and sex in the ventilatory responses of C57BL6 mice before and after HCC or HXC^136^ are somewhat understandable in that it could be the relative role of eNOS and associated functional proteins critical to ventilation may differ between sexes. Finally, the difference in the role of eNOS in C57BL6 mice compared to the 129/SV-C57BL6 hybrid mice^111^, support substantial evidence as to the importance of genetic/sex^86,90,92,96–101^ and neurochemical^86,91,102–107^ factors in ventilatory signaling in response to hypoxic and hypercapnic gas exposures.

The major strength of this and our companion study^136^ is the use of an established method (i.e., whole body plethysmography in freely-moving mice)^37,67–69,73,136,162^ to provide a detailed set of analyses about the role of sex in the differences in the ventilatory responses seen between WT and eNOS-/- C57BL6 mice that occurred during exposure to hypercapnic and hypoxic^136^ gas challenges and upon return to room-air. Aside from obtaining a deeper understanding of how hypoxic and hypercapnic challenges affect ventilatory timing and mechanics in male and female mice,  our studies provide a more complete understanding of the roles of eNOS in these processes than have been reported previously^71,84,85,111,112^. A major weakness of this study is that mice of C57BL6 background do not reflect what may happen in other species of mice (especially those that are less prone to ventilatory disturbances, such as apneas) and certainly in larger mammals, including humans^73,91–104^. Another weakness of this study is that we did not use other techniques, such as measurement of arterial blood-gas chemistry values (e.g., pH, pCO_2_, pO_2_, and sO_2_) and Alveolar-arterial gradient (i.e., the index of gas exchange within the lungs) to gain a deeper understanding of the role of eNOS in ventilatory control processes of male and female C57BL6 mice.

## Supplementary Information

Supplementary Information.
