# Supplementary material for: Ventilatory responses during and following hypercapnic gas challenge are impaired in male but not female endothelial NOS knock-out mice
Source: Sci Rep. 2021 Oct 18;11:20557. doi: 10.1038/s41598-021-99922-5 (PMC8523677; doi:10.1038/s41598-021-99922-5)
Supplement: Supplementary file 1 — Supplementary Information. [file 41598_2021_99922_MOESM1_ESM.docx]

**Ventilatory responses during and following hypercapnic gas challenge are impaired in male but not female endothelial NOS knock-out mice**

Paulina M. Getsy,^1,2^ Sripriya Sundararajan,^3,^* Walter J. May,^3^ Graham C. von Schill,^3^

Dylan K. McLaughlin,^3^ Lisa A. Palmer,^3^ Stephen J. Lewis^1,4,5,†^

*^1^Department of Pediatrics, Case Western Reserve University, Cleveland, OH, USA*

*^2^Department of Physiology and Biophysics, Case Western Reserve University, Cleveland, OH, USA*

*^3^Pediatric Respiratory Medicine, University of Virginia School of Medicine, Charlottesville, VA, USA*

*^4^Department of Pharmacology, Case Western Reserve University, Cleveland, OH, USA*

*^5^Functional Electrical Stimulation Center, Case Western Reserve University, Cleveland, OH, USA*

***Current Address:** Division of Neonatology, Department of Pediatrics, University of Maryland School of Medicine, Baltimore, Maryland 21201, USA

**^†^Correspondence:** Stephen J. Lewis, PhD. Department of Pediatrics, Biomedical Research Building BRB 319, Case Western Reserve University, 10900 Euclid Avenue Mail Stop 1714, Cleveland, Ohio 44106-1714. Email: sjl78@case.edu

**Acknowledgments**

The authors also wish to acknowledge the important contributions of Ms. Chelsea Csuhran and Ms. Yvonnda West to the data collection and data analyses. The authors also wish to thank the staff at the Animal Care Facilities at the University of Virginia for their care of the mice.

**Abbreviated Title (Running Head):** Hypercapnia response in eNOS knockout mice

**Keywords**

hypercapnia, C57BL6 mice, ventilatory parameters, endothelial nitric oxide synthase, carotid body

**Supplemental Table 1.** Resting parameters in male WT and eNOS-/- knock-out mice from this and our companion manuscript^136^

In the present manuscript “*Ventilatory responses during and following hypercapnic gas challenge are impaired in male but not female endothelial NOS knock-out mice*” and also in our companion manuscript entitled “*Short-term facilitation of breathing upon cessation of hypoxic challenge is impaired in male but not female endothelial NOS knock-out mice*”,^136^ we evaluated the changes in 11 ventilatory parameters, namely, frequency of breathing, tidal volume (TV), minute ventilation (MV), inspiratory Time (Ti), expiratory Time (Te), Ti/Te, inspiratory drive (TV/Ti), expiratory drive (TV/Te), peak inspiratory flow (PIF), peak expiratory flow (PEF) and PIF/PEF. There were no differences in baseline values between female wild-type (WT) and eNOS knockout (eNOS-/-) mice in the present study (hypercapnic challenge) and in the companion (hypoxic challenge)^136^ study. As shown **in the table below,** there were no differences in baseline values between the male WT and eNOS-/- mice in the hypoxia study, whereas in the hypercapnia study there were 4 parameters, TV, MV, expiratory drive (ExpD) and PEF that were lower in male eNOS-/- mice than male WT mice.

None of these differences could be explained by differences in body weights as they were very similar to one another (**Table 2**). In addition, there were relatively large numbers of mice in each group and the standard errors for each ventilatory parameter were less than 5% of the mean in every instance. We have no ready/obvious explanation why the resting values for these 4 parameters were lower in male eNOS-/- than male WT in the present (hypercapnia) study and not in the companion (hypoxia) study^136^ since in addition to the above points, there were no differences in (1) vendor (*Jackson Laboratories*), (2) age of the mice, (3) housing and feeding conditions, (4) time of year that the studies were performed, or (5) temperature and humidity of the room in which all of the studies were performed. Nonetheless, we do not believe that these findings compromised either study, especially since it was only the male eNOS-/- mice that displayed differences to their wild-type counterparts in the present (hypercapnia study), and not the female eNOS-/- mice in either the present (hypercapnia study) or the companion (hypoxia study)^136^.

**Supplemental Table 1.** Resting parameters in male WT and eNOS-/- knock-out mice

|  | **Hypercapnia Study** | |  | **Hypoxia Study** | |
| --- | --- | --- | --- | --- | --- |
| **Parameter** | **WT** | **eNOS-/-** |  | **WT** | **eNOS-/-** |
| Numbers | 11 | 13 |  | 12 | 13 |
| BW, gram | 25.3 ± 0.6 | 24.2 ± 0.6 (-4.4%) |  | 24.9 ± 0.5 | 23.7 ± 0.6 (-4.8%) |
| TV, ml | 0.214 ± 0.009 | 0.184 ± 0.005* (-14.0%) |  | 0.205 ± 0.005 | 0.196 ± 0.009 (-4.4%) |
| MV, ml/min | 39.8 ± 1.7 | 34.0 ± 1.3* (-14.6%) |  | 37.4 ± 1.3 | 35.6 ± 1.3 (-4.8%) |
| ExpD, ml/sec | 0.99 ± 0.05 | 0.80 ± 0.04* (-19.2%) |  | 0.92 ± 0.03 | 0.88 ± 0.03 (-4.4%) |
| PEF, ml/sec | 2.17 ± 0.13 | 1.67 ± 0.08* (-23.0%) |  | 2.97 ± 0.14 | 2.89 ± 0.12 (-2.7%) |

The data are presented as mean ± SEM. WT, wild-type C57BL6 mice. eNOS-/-, endothelial nitric oxide synthase knock-out mice. **P* < 0.05, eNOS-/- versus WT mice.

136. Getsy, P.M., Sundararajan, S., May, W.J., von Schill, G., McLaughlin, D.K., Palmer, L.A. & Lewis, S.J. Short-term facilitation of breathing upon cessation of hypoxic challenge is impaired in male but not female endothelial NOS knock-out mice. Sci. Rep. under review (2021).

**Supplemental Figure 1**

**A.**

**B.**

**C.**

**D.**

**E.**

**Supplemental Figure 1.** **Panels A and B:** Inspiratory time/Expiratory time (Ti/Te) ratio before, during a hypercapnic (HC) gas challenge (5% CO_2_, 21% O_2_, 74% N_2_) and upon return to room-air in male (M) and female (F) wild-type (WT) and eNOS knock-out (eNOS-/-) mice. **Panels C and D:** Arithmetic changes in Ti/Te ratio in male and female WT and eNOS-/- mice during the first 90 sec of exposure to the HC challenge and the first 90 sec upon return to room-air. **Panel E:** Total changes in Ti/Te ratio in male and female WT and eNOS-/- mice during HC challenge and during the first 5 min (RA5) and entire 15 min (RA15) return to room-air. The data are presented as mean ± SEM. *P < 0.05, significant change from pre-values. **^†^**P < 0.05, eNOS-/- *versus* WT within each sex.

**Supplemental Figure 2**

**E.**

**B.**

**A.**

**C.**

**D.**

**Supplemental Figure 2.** **Panels A and B:** Peak Inspiratory Flow/Peak Expiratory Flow (PIF/PEF) ratio before, during a hypercapnic (HC) gas challenge (5% CO_2_, 21% O_2_, 74% N_2_) and upon return to room-air in male (M) and female (F) wild-type (WT) and eNOS knock-out (eNOS-/-) mice. **Panels C and D:** Arithmetic changes in PIF/PEF in male and female WT and eNOS-/- mice during the first 90 sec of exposure to HC challenge and the first 90 sec upon return to room-air. **Panel E:** Total changes in PIF/PEF in male and female WT and eNOS-/- mice during the HC challenge and during the first 5 min (RA5) and entire 15 min (RA15) return to room-air. The data are presented as mean ± SEM. *P < 0.05, significant changes from pre-values. **^†^**P < 0.05, eNOS-/- *versus* WT within each sex.
